# 

**Published:** 1781-06

**Authors:** 


### SECTION IV.

### MONTHLY CATALOGUE.

1. ESSAYS on Phyſiological Subjects. By *J. Elliot*, M. D. 8vo. *Johnſon*, London, 1781. 48 pages, 1s. 6d.

The author of this ingenious tract is already known to the world by his *Philoſophical Obſervations on the Senſes of Viſion and Hearing*, &c. In the preſent performance he has proſecuted the the enquiry concerning animal motion and heat, which, among other ſubjects, he had treated of in his former work; and has alſo extended his reſearches to other branches of phyſiology.

Among other new matter we meet with the following remarkable facts:

In conſequence of an idea which he had ſuggeſted concerning the uſe of the bile, our author lived for ſome time wholly on bread and radiſhes; and afterwards for a longer time on mutton and beef. During the vegetable courſe bile ſeemed to abound leſs in the inteſtines and fæces than during the animal, and therefore probably employed more bile. To be ſatisfied of this, he digeſted, in half the gaſtric juice taken from a dog's ſtomach, ſome bread and radiſh, and in the other half ſome fat and lean mutton; then added to each a little of the animal's gall. The preparations were alike in both caſes; but the animal mixture taſted ſtrongly of the bile; the vegetable hardly at all: and though he afterwards doubled the quantity of bile in the latter, it ſtill ſeemed to taſte leſs of it than the other.

The author endeavours to deduce from theſe, and other experiments, the theory of the phlogiſtic and antiphlogiſtic regimens, and the uſe of the *bile* in the animal ſyſtem. His obſervations on on theſe ſubjects ſeem to unfold to us a new ſyſtem of phyſiology, and we cannot but recommend a peruſal of the work, as well as the proſecution of the ſubject, to the lovers of this important branch of medical ſcience.

2. Diſſertatio Medica Inauguralis de Podagra, auctore *Thoma Jeans*, Anglo, Societ. Med. Reg. Edin. Sod. et Soc. Chir. Med. Edin. S. H. 4to. Lugduni Batavorum, 1780. 25 pag.

3. L'art de ſoigner les pieds; contenant un traité ſur les cors, engelures, et les accidens des ongles et leur difformités; preſenté au roi, par *M. Laforeſt*, Chirurgien pedicure de S. M. et de la famille royale. *i. e.* The art of managing the feet; containing a treatiſe on corns, chilblains, and the diſeaſes and deformities of the nails. Preſented to the King, by *M. Laforeſt*, Corn-cutter to his Majeſty and the Royal family, 8vo. Paris, 1781.

4. Eſſai ſur l'action de l'Air dans les maladies contagieuſes, qui a remporté le prix propoſé par la Société Royale de Medecine. Par *M. J. J. Menuret*, Aſſocié regnicole de la meme Société. *i. e.* An Eſſay on the effects of the air in contagious diſeaſes; which obtained the prize offered by the Royal Medical Society. By *J. J. Menuret*,

*nuret*, one of the Home Members of the Society. 12mo. Paris, 1781. 136 pages.

5. L'art d'aſſayer l'or et l'argent; tableau comparé de la coupellation des ſubſtances metalliques, par le moyen du plomb et du biſmuth; procedé pour obtenir l'or le plus fin, &c. Par *M. Sage. i. e.* The art of aſſaying gold and ſilver; a comparative table of the cupellation of metallic ſubſtances by means of lead and biſmuth; a proceſs for obtaining the fineſt gold, &c. with plates. By *M. Sage.* 8vo. Paris, 1780. 122 pages.

6. Agri Romani hiſtoria naturalis, tres in partes diviſa; ſive methodica ſynopſis naturalium rerum in agro Romano exiſtentium, a *Philippo Aloyſio Gilio* concinnata. Pars prima, regnum animale. Tomus primus, ornithologia, in quâ de priori avium claſſe. 8vo. Romæ, 1781.

In this volume the author deſcribes only the granivorous birds, which conſtitute his firſt claſs. His deſcriptions, which appear to be extremely accurate, are illuſtrated by twenty-four engravings.

7. Pythographie economique de la Lorraine, ou Recherches botaniques ſur les plantes utiles dans les arts; ouvrage couronné dans la ſeance publique de l'Academie Royale des Sciences, Arts et Belles Lettres de Nancy, le 8 Mai 1779. Par *M. Willemet*, doyen des apothicaires, demonſtrateur royal de botanique et de chimie au college de medecine de Nancy, des academies de Lyon, Dijon, &c. *i. e.* The œconomical Pythography of Lorraine, or botanical inquiries relative to plants uſeful in arts; being the work crowned by the royal academy of ſciences, arts, and belles lettres at Nancy, at their public meeting on the 8th of May, 1779. By *M. Willemet*, maſter of the apothecaries company, regius demonſtrator of botany and chemiſtry in the college of phyſicians at Nancy, and member of the academies of Lyon, Dijon, &c. 8vo. Nancy, 1780. 142 pages.

8. Aſſemblée publique de la Societé Royale des Sciences, tenue dans la grande ſalle de l'hotel de cette ſocieté, en preſence des etats de la province de Languedoc, le 28 Decembre 1779. *i. e.* An account of the public meeting of the Royal Society of Sciences, held in the hall belonging to the ſociety, in preſence of the States of the Province of Languedoc, Dec. 28, 1779. 4to. Montpellier, 1780. 126 pages.

This collection contains the eulogies of Linnæus and the late Cardinal de la Roche Aymon; a paper by M. Gouan, on the cure of ſpecks in in the eyes; remarks on the Worabie, an Abyſſinian bird, by the Baron de Faugeres; on the regeneration of flat bones, by M. Vigarous; on the number of births and deaths at Montpellier, by M. Mourgue, who finds the proportion of deaths annually to be one in twenty-ſix; on the uſe of the milk of animals for foundlings, by M. Brun; on the electrical cauſe of earthquake, by M. Bertholon; and on the circulation of the air in mines, by M. Genſane, jun.

9. Carte mineralogique de France, où ſont marqués les differens terreins principaux qui partagent ce Royaume, et les ſubſtances particulieres qu'il renferme; dreſſée ſur les obſervations de M. Guettard, de l'academie des ſciences. Par *M. Dupain Triel* pere, Geographe du Roi et de Monſieur. *i. e.* A mineralogical map of France, in which are marked the principal varieties of ſoil that occur in that kingdom, together with the particular ſubſtances included in it; founded on the obſervations of M. Guettard, of the academy of ſciences. By *M. Dupain Triel*, ſen. Geographer to the King and to Monſieur. Paris, 1781. price 2 livres, 8 ſols.

10. Die natürliche magie aus allerhand beluſtigenden und nützlichen kunſtſtücken beſtehend, hend, zuſamenn getragen. Von *J. C. Wiegleb. i. e.* Natural magic; being a collection of uſeful and entertaining performances. By *J. C. Wiegleb.* 8vo. Berlin, 1779. 416 pages, with copper-plates.

This is a very rational and amuſing compilation.

11. *J. A. E. Götze* entomologiſche beyträge zu des R. Linnée 12ten. auſgabe des natur-ſyſtems. *i. e.* Entomological ſupplements to the 12th edition of Linnæus's ſyſtema naturæ. By *J. A. E. Götze.* Vol. I. Part III. 8vo. Leipſic, 1780. 390 pages.

INDEX.

### I N D E X.

A.

Page

AASKOW, U. B. on an epidemic ſcarlatina, — 379

———— hepatitis, — — 391

Abildgaard, P. C. his account of a tertian, — 395

Abdomen, abſceſs of, — — 328

Acta, Havnienſia, — — 377

Aery, Dr. Thomas, his remarks on hydrocephalus internus, — 424

Air, obſervations on, — — 51

—— fixed, remarks on, — — 56, 440

Aitken, John, outlines of the theory and cure of fever, — 366

Amar, Don Joſeph, on the ſmall pox, — — 72

———— eruptive diſeaſes, — 142

———— diſeaſes of the breaſt, — 143

Amenorrhœa, experiments in, — — 92

Andree, John, account of an elaſtic trochar, — — 418

Andry, M. remedy diſcovered by him for the gonorrhœa, — 433

Aneuriſm, ſpurious, caſe of, — — 334

Angina trachealis, treatiſe on, — — 217

Antiſpaſmodics, remarks on, — — 12

Applications, warm, bad effects of in wounds of the head, — 324

Arm, fractured, remarkable operation on, — — 356

Aſcites, cure of, — — — 263

Aſphaltum, oil of, recommended in phthiſis, — 397

Aſafœtida, its uſe in carious ulcers, — — 239

Aſti, Felix, on the poiſon of mad animals, — 370

B.

Bang, F. L. on the angina and ſcarlatina, — — 379

———— putrid fever, — — 384

———— the connexion of intermittents with other diſeaſes, 389

———— oleum aſphalti, — — 397

Baſtays, M. his theory of chronic diſeaſes, — — 212

Bath, warm, recommended in morbid irritability, — 182

Bayley, Richard, on angina trachealis, — — 217

Bergman, Sir Thorbern, his opuſcula phyſica, — 51, 73

———— French tranſlation of, — 293

Berkley, J. F. Van, natural hiſtory of Holland, — 373

Bingers, M. of a ſuppuration of the omentum, — 241

———— wound happily healed, — — 242

———— the uſe of lime water and ſoap in calculus, — *ibid.*

———— a caſe of a worm perforating the inteſtines, — 243

———— tumour near the rectum, — *ibid.*

———— fiſtula in perinæo, — — *ibid.*

———— an excreſcence in the rectum, — 244

———— a cancerous teſticle, — — 245

———— the uſe of cold water in wounds of the joints, *ibid.*

Black, Dr. W. on the ſmall pox, — — 295

Blackall, Andrew, preparations in his muſeum, — 359

Blackburne, Dr. Thomas, of hydatids voided with the urine, 125

Bland, Dr. his reſearches on population, &c. — 355

Blane, Dr. Gilbert, his account of a hurricane, — 132

L l l    Elizard,

Page

Blizard, William, on the fistula lachrymalis, — 62

Block, M. on the use of asafœtida in caries, — 239

——— of a man who swallowed pins, &c. — — 334

Born, M. his mineralogical travels, — — 213

Brain, concussion of, — — 324

——— suppuration of, — — — ibid.

——— wound of by a musket ball, — 325

Bread, fresh baked, ill effects of, — — 333

Breasts, abscesses of how to be treated, — — 167

Brownrigg, Dr. his discovery relative to air, — 276

Bruno, Joh. elementa medicinæ, — — 68

Buxton, accounts of, — — 276, 434

C.

Calamus aromaticus, its efficacy in intermittents, — 388

Calendarium medicum, — — 213

Calmé, M. on generation, — — — 292

Callisen, H. of excessive intoxication, — — 396

——— several remarkable cases, — — 399

Cancer, ointment for, — — 193

Cantharus, a Swedish measure, described, — — 75

Carbuncle, of Burgundy, dissertation on, — — 370

Caries, of the spine, case of, — — 271

——— scull, — — — 334

Catamenia, *see* Menses, — —

Cavallo, Tiberius, on medical electricity, — 246

Cavear, how prepared, — — — 26

Cazelles, M. de, on medical electricity, — — 291

Changeux, M. meteorographie, — — 215

Children, management of, observations on, — — 171

——— new born, diseases of, — — 172, 408

Clare, Peter, on the gonorrhœa, — — 368

Clark, Thomas, case of epilepsy, — 428

Cold, experiments on, — — 433

Colic, occasioned by a piece of iron in the intestines, — 327

Collin, H. on lactuca sylvestris in dropsy, — — 263

Consumption, venereal, cured by frictions, — 129

Corns, treatise on, — — —

Costiveness, case of, — — 349

Cough, purulent, cured by an abscess, — — 386

Cramer, M. of a spurious aneurism, — — 334

Crawford, Dr. examination of his theory of heat, — 109

Cremadells, F. nova physiologia, — — 295

Cullen, Dr. William, German translation of his first lines, 141

Czenpinsky, P. dissert. zoologico-medica, — — 68

D.

Deaths, lists of, — — 66, 137, 209, 282, 363, 438

Diabetes, remarks on, — — 89

Diarrhœa, cured by verbascum, — — 96

Dibel, M. of a suppuration of the omentum, — 336

Diet, remarks on, — — — — 411

Dissection, accounts of, 9, 11, 89, 128, 203, 205, 218, 219, 222, 225, 271, 283, 309, 322, 325, 383, 400, 401, 423

Dropsy, experiments in, — — 91

——— cured by the lactuca sylvestris, — 264

Page
Dropfy, cured by blue vitriol, — — 266
Duhaume, M. on domeftic remedies, — — 71
Duncan, Dr. Andrew, heads of lectures, — — 68

E.

Ears, their confent with the lungs, — — 177
Eau de Luce, method of preparing it, — — 193
Ebart, F. C. G. de obefitate, — — 293
Eichel, J. of an epidemic fcarlatina, — 377
Elbow joint, fingular difeafe of, — — 329
Electricity, medical, remarks on, — 246, 291, 374
Elliot, Dr. John, of an obftinate coftivenefs, — 349
—————— phyfiological effays, — — 438
Emerigon, M. his fpecific for the gout, — 210
Emmenagogues, none that can be relied on, — 147
Epilepfy, cafe of, — — 428
Euers, M. on herpes, — — — 240
Eye, purulent, remarks on, — — 115

F.

Falconer, Dr. William, on the influence of climate, &c. — 409
Feet, treatife on the management of, — — 440
Fever, miliary, remarks on, — — — 168
——— puerperal, — — 9, 169, 408
——— putrid, — — — 384
——— outlines of the theory and cure of, — 366
Fire, obfervations on, — — — 312
Fiftula in perinæo, cafes of, — — 320, 379
Flooding, uterine, how to reftrain, — 59, 148, 155
Fluor albus, remarks on, — — — 151
Fœtus, curious mifplacement of the vifcera in one, — 205
——— head of, feparated in the uterus, — 384
Foot, Jeffe, his critical inquiry, — — 210
Ford, Edward, on the cure of hydrophthalmia, — 346
Fofter, Dr. Edward, treatife on midwifery, — 402
Fothergill, Dr. John, an affectionate tribute to the memory of, 210
Foujols, M. on hernias — — 291
Fractures, machine for, — — 239
Fuefsly, J. C. magazine for lovers of entomology, — 69
Furunculus, cafe of, — — — 332

G.

Geifler, M. of a wound of the head, — 323

Gellert, C. E. his metallurgical chemiftry, — 140

—————— art of affaying, — — 141

Generation, effay on, — — — 292

Gerbier, M. effects of his remedy for cancers, — 206

Giefeman, M. of a delivery retarded by a prolapfus vaginæ, — 335

Gilio, P. A. agri Romani hiftoria naturalis, — 441

Gleditch, J. G. on fimple medicines, — — 373

Goducke, M. cafes by, — — 329

Götze, J. A. E. entomological fupplements to Linnæus, 444

Gonorrhœa, treatifes on, — — 215, 368

Goulin, M. on the time at which Afclepiades flourifhed, — 71

Gout, fpecific for, — — 210

——— obfervations on, — — 199

Guerin, M. on the gonorrhœa, — — 215

Page

Gulbrand, J. W. of a purulent cough, — 386

——— practical remarks by, — — 387

——— of the efficacy of an issue in ophthalmia, — 398

——— morbus maculosus hæmorrhagicus, — 382

H.

Hagen, M. of a mortification of the vagina, — 335

——— prolapsus of the colon, — *ibid.*

Haller, Albert, new edition of his primæ lineæ, — 372

Hamilton, Alexander, treatise of midwifery, — 145

Hæmorrhage, uterine, treatment of, — — 59

——— fatal, from the anus, — — 382

Hayes, Thomas, on Buxton waters, — — 434

Hellebore, useful remarks relative to, — 150

Hemiplegia, case of, — — 323

Henbane, common, its fatal effects, — — 338

Hepatitis, remarks on, — — 391

Herbert, J. de, on electricity, — — 374

Hernias, advice concerning, — — 292

——— cases of, — — 240

Herpes, remarks on, — — 94

Hints, on diseases that are not cured, — 364

Hird, Dr. William, his affectionate tribute, &c. — 210

Home, Dr. Francis, clinical experiments, — 1, 85

Horn, M. on fresh baked bread, — — 333

Hubert, M. citoyen dentiste, — — 144

Hurricane, curious effects of one, — 133

Hydatids, voided with the urine, — — 125

Hydrocephalus internus, new mode of treating it, — 357

——— use of mercury in, — 358, 424

Hydrophobia, facts relative to, — 370

Hydrophthalmia, case of, — — 346

Hypochondriasis, remarks on, — — 398

Hysteria, cured by the bark, — — 383

J.

Jackson, Dr. Seguin Henry, treatise on sympathy, — 284

Jadelot, N. his physiology, — — 372

Jayant, M. le, his grand remedies, — — 143

Jeans, Thomas, de podagra, — — 440

Intermittents, experiments in, — — 2

——— their connexion with other diseases, — 389

Intoxication, excessive, case of, — — 396

Isinglass, how prepared, — — 25

Issue, the efficacy of one in ophthalmia, — 398

Jung, M. of a dangerous wound, — — 331

K.

Keyser, M. baneful effects of his pills, — — 206

Kirwan, Richard, his chemical researches, — 65

——— his notes on Scheele, — — 312

Koelpin, Alex. of a fistula in perinæo, — 379

——— B. on the rhododendron chrysanthum, — 226

Kohler, M. his account of a concussion of the brain, — 324

Kuhn, M. of a suppuration of the leg, — — 330

——— an inflammatory sore throat, — *ibid.*

### INDEX.

Page

L.

Lactuca sylvestris, its efficacy in dropsy, — 263

Lambon, M. on the section of the symphysis putris, — 72

Lafond, M. dictionary of natural philosophy. — — 298

Laforest, M. on the management of the feet, — — 440

Laudun, M. on broths in fevers, — — — 71

Lauro-cerasus, experiments with, — — — 343

Leather, Russian, manner of preparing it, — — 23

Leeches, historico-practical essay on, — — 237

——— use of in hypochondriasis, — — 399

Lefevre, M. conjectures concerning his remedy for the gout, — 201

Leprosy, particular kind of, — — — 28

Le Roux, M. on canine madness, — — — 141

Leurye, M. de, on the Cæsarean section, — — 216

Lieutaud, M. anecdotes of, — — — 279

Lip, swelling of, a symptom of worms, — — 95

Lithonthriptics, remarks on, — — — — 97

Liver, obstruction of cured by mercury, — — — 390

Lues venerea, how to be treated in children, — — 388

Lumbago, remedy for, — — — — 87

Lungs, wound through, — — — — 328

M.

Magnesia, remarks on, — — — 83

Manning, Dr. Henry, his modern improvements in physic, — 68

——————————————— surgery, 139

Map, mineralogical, of France, — — — 443

Maret, M. eulogy of, — — — — — 371

——— on the small pox, — — — 223

Martinet, M. on volatile alkali, — — — — *ibid.*

Maupin, M. course of chemistry, — — — — 290

Melæne, remarks on, — — — 10

Mellins, C. F. materia medica, — — — 294

Menses, final cessation of, with what symptoms attended, 150, 175

——— their influence on health, — — — 174

——— remedies in obstruction of, — — — 92, 147

Menstruation, painful, how to obviate, — — — 148

Menuret, J. J. on the effects of air, — — 440

Metastasis, remarks on, — — — — 178

Meza, S. T. de, on a venereal consumption, — — 129

——————— the malignant scarlatina — — 379

——————— cure of an ascites, — — — 394

——————— his account of a death occasioned by the force of imagination, — — — — — — 396

Midwifery, treatises on, — — — — 145, 402

Monro, Dr. Alexander, his works — — — 297

——————————— life, — — — — 298

Montpellier, Assemblée publique de la societé de, — 442

Morgan, William, on heat, — — — 109

Music, instances of its good effects, — — — 183

N.

Napellus cæruleus, its efficacy in rheumatism denied, — — 341

Nicola, E. A. his pathology, — — — 214

Nerves, disorders of, treatise on, — — 32, 98, 174

O. Omentum,

### I N D E X.

Page

O.

Omentum, suppuration of, — — — 241, 336

——— dropsy of, — — — — 422

Ophthalmy, remarks on, — — — — 115

Opium, use of in intermittents, condemned, — — 136

Ovarium, singular tumour of, — — — 394

——— curious appearance in one on dissection, — — 203

P.

Pallas, P. S. his travels, — — — 20, 185

Palsy, experiments in, — — — — 85

Pharmacopœia, Brunsvicensis, — — — 189

Phosphorus, effects of in fevers, — — — 208

Phthisis, experiments in, — — — 9

Physic, modern improvements in the practice of, — — 68

Pia, M. on the recovery of drowned persons, — — 372

Pistor, M. of a fatal wound of the forehead, — — 324

Planque, M. his medical observations, — — — 142

Plenk, I. I. de morbis venereis, — — — 70

Poisons, remarks on, — — — 38, 336

Polypus of the nose, case of, — — — — 321

Pott, I. H. anecdotes of, — — — — 137

Pregnancy, remarks on, — — — — 152

——— symptoms of, — — — 154, 405

Priestley, Dr. Joseph, French translation of his experiments on air, 142

Promotions, — — — 66, 208, 281, 362, 437

Psorophthalmy, remarks on, — — — 115

Pubis, symphysis, section of, — — 72, 134

Q.

Quassia, its efficacy doubted, — — — —

Questions, prize, 64, 132, 203, 274, 275, 278, 354, 384, 431

R.

Rahn, J. H. adversaria medico practica, — — 294

Ramdohr, M. case of a gun-shot wound, — — — 325

Recovery, unexpected, three cases of, — — 397

Reflexions, for young physicians, &c. — — 214

Rheumatism, remedy for, — — — 226

Riediger, M. of a luxation of the vertebræ — — 327

Riesenbach, M. case of a hand torn off by a mill, — — 325

——— of a spitting of blood, — — 335

Rogert, J. P. on retroverted uterus, — — — 392

Roufferie, M. de, on the waters of Jaleyrac, — — 369

S.

Sage, M. on assaying gold and silver, — — — 441

Sanberg, M. on the water of Spa, — — — 294

Saponaria, officinalis, a remedy for gonorrhœa, — — 433

Saxtorph, M. on uterine hæmorrhages, — — — 59

——— a tumour of the ovarium, — — 394

——— stricture of the uterus, — — 384

Scarlatina, anginosa, account of, — — — 377

Scarpa, A. Anatomicæ annotationes, — — — 211

Scheele, C. W. on air and fire, — — — 312

Schoenheider, H. on a cure of hysteria by the bark, — — 383

——— the venereal disease in children, — 388

——— instances of unexpected recovery, — 397

Schoenheider,

Page
Schoenheider on hypochondriafis, — — — 398
Schopper, M. of a furunculus, — — — 332
Schmucker, J. L. furgical effays — — 231, 320
— — on amputations, — — — 231
— — the furunculus, — — — 332
— — leeches, — — — — 237
Schumacher, M. feveral cafes in furgery, — — 320
Sciatica, experiments in, — — — 86
Scopoli, J. A. fundamenta chemiæ — — 69
Sellie, M. of a luxation of the vertebræ, — — 326
— — caries, — — — 334
Senfibility, morbid, inftances of, — — 177
Seton, ufe of in the hydrophthalmia, — — — 348
— — epilepfy, — — — 4 8
Simmons, Dr. Samuel Foart, German tranflation of his anatomy, 141
— — new edition of his elements of anatomy, 367
— — on a caries of the fpine, — 271
Sims, Dr. James, editor of Dr. Fofter's midwifery, — — 492
Smeathman, Henry, profpectus of his travels — — 359
Spa, effay on the waters of, — — — — 224
Sponitzer, M. of a colic from bone in the inteftines, — 327
— — wound in the lungs, — — 328
— — an abfcefs of the abdomen, — — ibid.
Societies, meteorological, eftablifhment of, — — 432
Stimuli, diftinctions of, — — — — 176
Sugar, acid of, remarks on, — — — 81
Surgery, modern improvements in, — — — 139
Swediar, Dr. F. his cafe of a white fwelling, — — 194
Sympathy, treatife on, — — — 284

T.

Tartar, cream of, its efficacy in dropfy, — — 91

Tertian, cafe of, with uncommon fymptoms, — — 395

Teftes, their fituation and defcent in the fœtus, — 376

Thebaic tincture, its efficacy in ophthalmy, — — — 119

Theden, I. A. his defcription of a machine for fractures, 238

Tibia, fiffure of, — — — — 329

Tiffot, M. on difeafes of the nerves, — — 32, 98, 174

Tode, J. C. of an obftruction of the liver, — — 390

Tranfactions, Philofophical, German tranflation of, — 375

Trichiafis, curious cafe of, — — — 121

Trochar, elaftic, account of, — — — 418

Tumour, fteatomatous, fingular one in the thorax, — — 387

— — kidney, — 388

Typhus nervofus, experiments in, — — — 4

U.

Vagina, mortification of, — — — 336

— prolapfus of hindering delivery, — — — ibid.

Vaftapani, P. I. on the Peruvian bark, — — 144

Vegetables, poifonous, obfervations on, — — — 336

Venæfection, inftances of its abufe, — — 179

Verbafcum, its efficacy in diarrhœa, — — — 96

Vertebræ, diflocation of, — — 326, 327, 401

Vetillard, M. on the dyfentery, — — — 144

Villers, M. profpectus of his work on infects, — — 133

Vinegar,

Page

Vinegar, not an antidote to arfenic, — — — 136

Urine, incontinence of, how cured, — — — 135

Uterus, fingular ftricture of, — — — 384

——— retroverted, cafe of, — — — 392

W.

Ware, James, on the ophthalmy, — — — 115

Water, fea, curious remarks on, — — 75

——— mineral, methods of imitating, — — ibid.

Weed, fever fo called, what, — — — 168

Wiegleb, J. C. natural magic, — — 444

Wilmer, B. on vegetable poifons, — — — — 336

Willemet, M. Pytographia de la Lorraine, — — 441

Wilfon, Mr. his experiments on cold, — — 433

Wright, Dr. William, on the efficacy of blue vitriol in dropfy, 266

Wrifberg, H. A. anatomical differtations, &c. — 375, 376

Wurm, M. of an hemiplegia, — — — 323

Wurtz, G. C. mappa medicamentorum, — — — 70

Z.

Zinn, J. G. de oculo humano, — — — 291

